# Risk factors that affect the degree of bronchopulmonary dysplasia: Comparison by severity in the same gestational age

**DOI:** 10.1371/journal.pone.0235901

**Published:** 2020-07-16

**Authors:** Sung-Ha Kim, Yea Seul Han, Jiyoung Chun, Myung Hee Lee, Tae-Jung Sung

**Affiliations:** 1 Department of Pediatrics, Kangnam Sacred Heart Hospital, Hallym University Medical Center, Seoul, Korea; 2 Division of Neonatology, Department of Pediatrics, Samsung Medical Center, Seoul, Korea; 3 Department of Statistics and Data Center, Samsung Medical Center, Seoul, Korea; University of Oklahoma, UNITED STATES

## Abstract

**Objective:**

To investigate the risk factors for BPD severity by gestational age (GA) and identify a way to reduce the incidence of moderate-to-severe BPD

**Study design:**

This was a retrospective cohort study of very-low-birth-weight-infants (VLBWIs) delivered at 24 to 28 weeks GA from Korean Neonatal Network registry between 2013 and 2016. BPD was defined using the National Institutes of Health criteria. Study populations were divided by GA and subdivided into no/mild BPD and moderate/severe BPD. The initial statuses of all infants, including those who died before BPD diagnosis and the maternal and neonatal factors of the live infants were compared. Statistical methods included descriptive statistics, comparative tests, and logistic regression.

**Results:**

Of 3,976 infants, 3,717 were included (24weeks, n = 456; 25 weeks, n = 650, 26 weeks, n = 742; 27 weeks, n = 836; 28 weeks, n = 1,033). The overall mortality rate was 18% and the rates by GA were 43%, 29%, 11%, and 6% in the 24-, 25-, 26-, 27-, 28-GA groups, respectively. Small for GA (SGA), treated patent ductus arteriosus (PDA), hypotension, and late-onset sepsis were significant risk factors for developing moderate/severe BPD in the 25 to 28-week GA groups in the multivariate analyses. However, for infants born at 24 weeks GA, there were no significant risk factors apart from initial resuscitation.

**Conclusions:**

Effective initial resuscitation was the most important factor for infants delivered at 24 weeks GA determining the severity of BPD. For infants delivered between 25 and 28 weeks, judicious care of SGA infants, aggressive treatment for PDA and hypotension, and intense efforts to decrease the sepsis rate are needed to reduce the development of moderate-to-severe BPD.

## Introduction

Bronchopulmonary dysplasia (BPD) is an important medical condition that causes morbidity and mortality among surviving very low birth weight infants (VLBWIs) [1, 2]. It is also a chronic respiratory disease related to lung injury in preterm infants that leads to various long-term complications in terms of respiratory, cardiovascular, and neurological development from early childhood to adulthood [3, 4].

Advances in the field of neonatology have increased the survival rates of preterm infants. In addition, early surfactant administration with less invasive ventilation techniques in these populations has made it possible to reduce the amount of oxygen given to infants at the time of BPD assessment [5]. However, the incidence of BPD is still increasing or stable due to the increased survival rate of extremely preterm infants, even though the incidence of BPD is decreasing in preterm infants delivered at >28 weeks [6].

Due to the efforts of neonatologists to not only diagnose the condition but also manage the long-term complications, the definition and the severity of BPD attracted the attention of pediatricians. The first definition was proposed by Northway et al [7] over 50 years ago, and then it was revised by the National Institute of Child Health and Human Development (NICHD) in 2000, The new definition considered infants ≤32 weeks and >32 weeks postmenstrual age (PMA) and proposed a severity classification (mild, moderate, severe) [8].

There have been attempts and studies to explore the possible pathophysiology and actual risk factors for the development of this multifactorial disease; however, apart from GA and/or birth weight, few studies have investigated risk factors, such as prenatal conditions, initial stages or different postnatal factors related to ventilation, that could affect the severity of BPD [9–11].

Hence, the aim of this study was to identify risk factors that could affect BPD severity among infants of the same GA and, based on this information, to determine possible ways to reduce the incidence of moderate-to-severe BPD.

## Materials and methods

The Korean Neonatal Network (KNN), which was established by the Korean Society of Neonatology and Korea Centers for Disease Control and Prevention in 2013, collects nationwide population-based data regarding VLBWIs who have been admitted to the neonatal intensive care unit (NICU) [12]. There were 8,379 VLBWIs registered in the KNN registry between January 2013 and December 2016. Of those infants, 3,976 VLBWIs born from 24^+0^ to 28^+6^ weeks were examined in this study. The KNN registry was approved by the institutional review board at each participating hospital, including the Hallym University Medical Center (IRB number 2015-05-063) and informed written consent was obtained from the parents at enrollment by all the NICUs participating in the KNN. All the data are monitored regularly by KNN data management committee and the present study was approved by the KNN data management committee.

**Fig 1:**
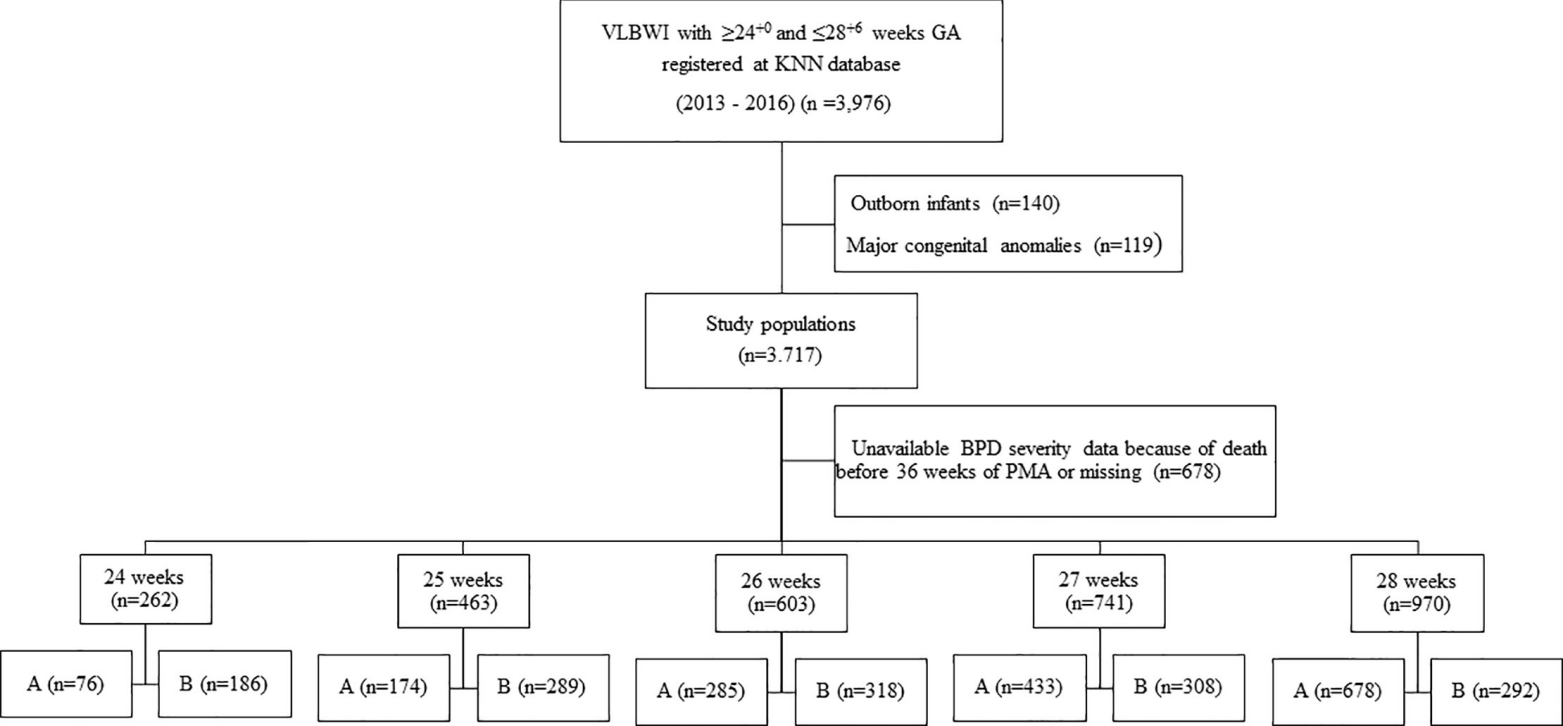


The exclusion criteria were infants with congenital anomalies (n = 119), and those who were born in another facility and transferred to the KNN registered institution (n = 140). After excluding those infants, we analyzed the initial status of the infants who were admitted to the NICU. Since there could be variable situations and different policies for the initial management protocol, we first analyzed the overall resuscitation status according to GA. Then, risk factors were investigated after excluding infants who expired before the BPD diagnosis. BPD was defined as a medical condition requiring supplemental oxygen at 36 weeks PMA and then we categorized the study infants as mild, moderate, or severe according to the NICHD criteria. First, we categorized the infants by their GA at 24 weeks, 25 weeks, 26 weeks, 27 weeks and 28 weeks. Then, the groups were subdivided into the no/mild BPD group (A) and the moderate/severe BPD group (B) according to BPD severity (Fig 1). After the first analysis of initial resuscitation status, infants who died before BPD diagnosis were excluded from the risk factor analysis.

Maternal factors were evaluated to investigate substantial effects on the severity of BPD according to GA. Antenatal data including multiple birth, oligohydramnios, complete antenatal steroids within 7 days of delivery, and delivery type were compared. Initial stepwise resuscitation measures, including positive pressure ventilation (PPV), intubation, chest compression, and epinephrine administration, that were performed immediately at delivery were analyzed. Features suspected to affect or have a relation with BPD severity, such as GA, birth weight, small for GA (SGA), sex, Apgar scores at 1 min and 5 min, air leakage, pulmonary hemorrhage, hypotension, surfactant use, patent ductus arteriosus (PDA), necrotizing enterocolitis (NEC, ≥ stage 2), intraventricular hemorrhage (IVH, ≥ grade 3), periventricular leukomalacia (PVL) and late-onset sepsis, were compared between group A and group B.

The definitions for the collected data were guided by the manual of the KNN. PPV was defined as positive pressure provided via a self-inflating bag, flow-inflating bag, or T-piece for any time duration. Complete antenatal steroids were defined as steroids given in the 7 days before delivery. Treated PDA (tPDA) was defined as PDA with medical and/or surgical treatment because of hemodynamically significant left-to-right shunt flow proven by echocardiogram and/or a pre/post saturation difference. Hypotension was defined as low blood pressure requiring inotropic medication during the first 7 days of birth. IVH and PVL were defined using Papile’s criteria by cranial ultrasonography [13] and the worst grade result during hospitalization was considered. NEC was defined according to Bell’s criteria (≥stage 2) [14]. SGA was defined as a birth weight within the 10^th^ percentile for GA based on a sex-specific growth chart [15]. Late-onset sepsis was defined as culture-proven bacteremia occurring after 3 days of age.

For statistical analyses, continuous variables were expressed as the means ± standard deviations, and categorical variables were expressed as numbers and proportions. Univariate analyses of categorical variables were performed with χ^2^ tests, and t-tests were used for continuous variables. Univariate and multivariate logistic regression were performed to identify the risk factor associated with the severity of BPD in individual GA groups. Multiple logistic regressions were performed for individual GA groups to estimate the odds ratio (OR) with 95% confidence interval (CI). The fit of the models was verified with the Hosmer-Lemeshow goodness-of-fit test. Statistical analyses were performed using SPSS version 24.0 (IBM Corp., Amarok NY, USA). A *P* value less than 0.05 was considered statistically significant.

## Results

During the study period, we evaluated 8,379 VLBWIs from the KNN database. Of them, 3,976 infants were born between 24^+0^ weeks and 28^+6^ weeks GA. Based on exclusion criteria, namely, out-born infants (n = 140) and infants with major congenital anomalies (n = 119), 3717 infants were enrolled in the study cohort (Fig 1). Of these infants, 456 (12.1%) were 24 weeks GA, 650 (17.5%) were delivered at 25 weeks GA, 742 (20.0%) were delivered at 26 weeks GA, 836 (22.5%) were delivered at 27 weeks GA, and 1033 (27.8%) were delivered at 28 weeks GA.

Regarding resuscitation status in the delivery room, the lower the GAs of the infants, the more likely they were to need step-by-step resuscitation. While intubation was needed in 98% of infants at 24 weeks GA, only 77% needed intubation at 28 weeks GA. Chest compression was needed in 49 out of 456 infants at 24 weeks GA (11%) and 37 out of 1033 infants at 28 weeks GA (4%).

The mortality rate decreased with increasing GA: 43% at 24 weeks GA, 29% at 25 weeks GA, 19% at 26 weeks GA, 11% at 27 weeks GA, 6% at 28 weeks GA (Table 1).

**Table 1:** Comparison of initial neonatal resuscitation at delivery room and initial status within an hour after admission to NICU (n = 3,717).

	24 weeks	25 weeks	26weks	27 weeks	28 weeks	Total
	(n = 456)	(n = 650)	(n = 742)	(n = 836)	(n = 1033)	(n = 3,717)
Resuscitation with PPV (%)	443(97)	625(96)	720(97)	762(91)	899(87)	3449(93)
Resuscitation with intubation (%)	445(98)	617(95)	681(92)	704(84)	797(77)	3244(87)
Resuscitation with compression (%)	49(11)	57(9)	54(7)	42(5)	37(4)	239(6)
Resuscitation with epinephrine (%)	31(7)	58(9)	39(5)	29(4)	18(2)	175(5)
Mortality, %	194(43)	187(29)	139(19)	95(11)	63(6)	678(18)
Apgar Score at 1 min.	3.0±1.6	3.6±1.7	3.8±1.8	4.3±1.8	4.7±1.8	4.1±1.8
Apgar Score at 5 min.	5.5± 1.9	6.1±1.8	6.3±1.7	6.6±1.6	6.9±1.5	6.5±1.7
Apgar Score at 5 min (≤3) (%)	140(31)	145(22)	129(17)	102(12)	82(8)	598(16)

Abbreviations: NICU, neonatal intensive care unit; GA, gestational age; PPV, positive pressure ventilation

**Fig 2:**
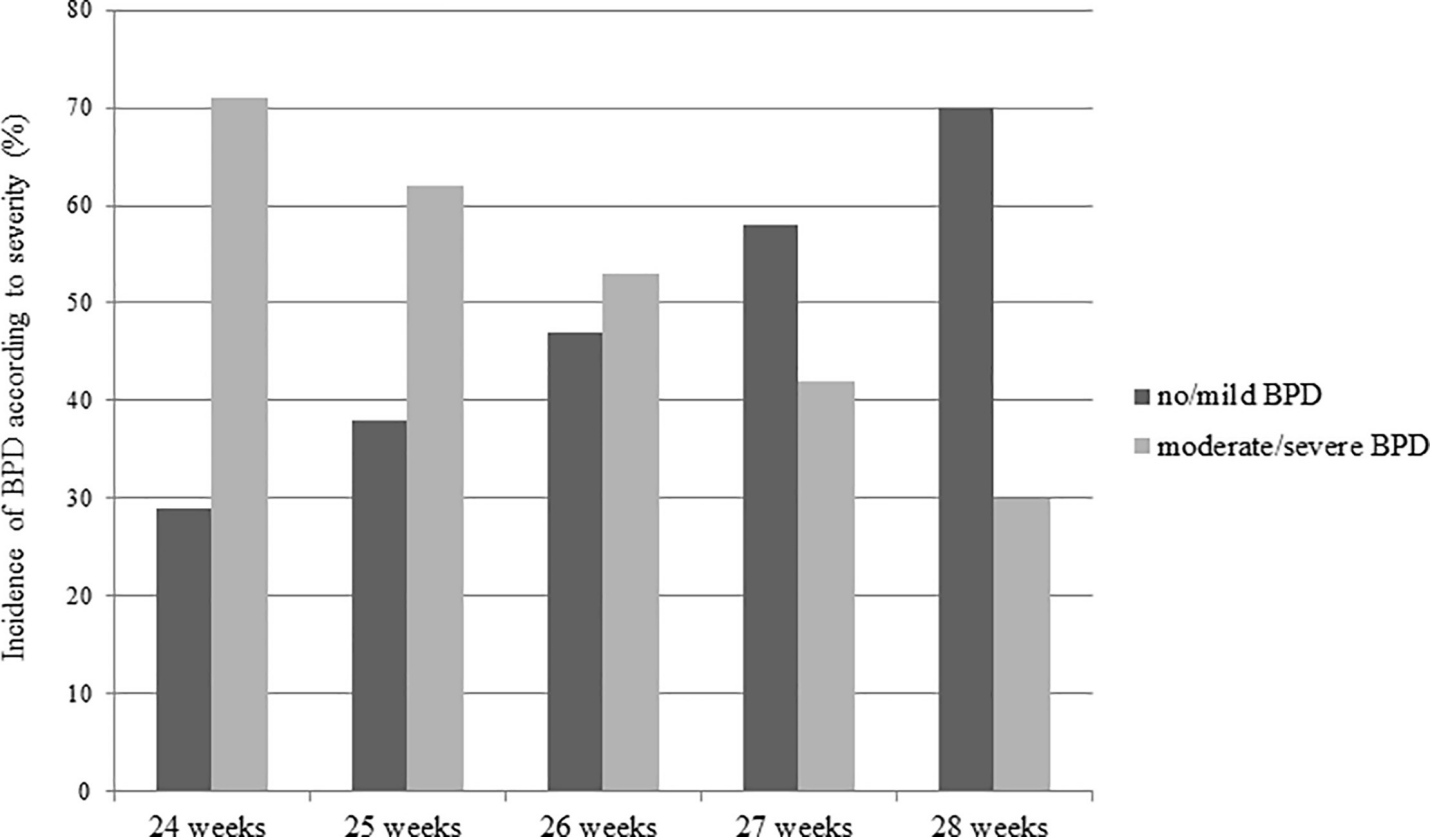


Since the aim of this study was to evaluate the risk factors for BPD severity, we excluded infants who died before discharge and those who had an unknown BPD severity (n = 678). Hence, 3,039 infants were enrolled for final analysis: 262 infants in the 24 weeks GA, 463 infants in the 25 weeks, 603 infants in the 26 weeks, 741 infants in the 27 weeks and 970 infants in the 28 weeks. Infants born at 24 weeks GA had the highest incidence of moderate-to-severe BPD (76/262, 71%) and the incidence decreased according to increasing GA to as low as 30% (292/970) in the 28 weeks GA group (Fig 2).

Although the analysis controlled for GA, a well-known strong risk factor for BPD, infants in the 26-week GA group showed a significant difference in the number of infants in the no/mild group (26.5±2.0 weeks) and moderate/severe group (26.4±2.0 weeks) (*P* = 0.004). In the 24-week GA groups, the birth weights of the infants were not different between the no/mild (697.3±108.7 grams) and moderate/severe groups (693.6±103.9 grams) but there were significant differences in birth weight between these subgroups in the rest of the groups (Table 2). The rate of SGA was significantly higher in the moderate/severe BPD group than in the no/mild group in the 25 weeks GA (9% *vs* 13%, *P* = 0.006), 26 weeks GA (8% *vs* 15%, *P* = 0.021), 27 weeks GA (6% *vs* 16%, *P* < 0.001), and 28 weeks GA (6% *vs* 20%, *P* < 0.001) but not different between the groups in 24-week GA groups (8% *vs* 10%, *P* = 0.562). There were no significant differences in the rates of multiple birth, oligohydramnios, and complete antenatal steroid use within 7 days of delivery between the no/mild and the moderate/severe GA groups. The rate of cesarean section was higher in the moderate/severe BPD group (83%) than in the no/mild group (76%) in the 28-weeks GA group (*P* = 0.030) while in the other GA groups, there were no significant differences.

**Table 2:** Comparison of demographic and maternal characteristics according to gestational age who survived during NICU stay (n = 3,039).

	24 weeks (n = 262)			25 weeks (n = 463)			26 weeks (n = 603)			27 weeks (n = 741)			28 weeks (n = 970)		
	A*	B**	*P*	A	B	*P*	A	B	*P*	A	B	*P*	A	B	*P*
	(n = 76)	(n = 186)		(n = 174)	(n = 289)		(n = 285)	(n = 318)		(n = 433)	(n = 308)		(n = 678)	(n = 292)	
GA, weeks	24.4±1.9	24.4±1.8	0.375	25.3±2.0	25.3±1.8	0.551	26.5±2.0	26.4±2.0	0.004	26.3±2.0	26.3±1.9	0.764	28.3±2.0	28.3±1.9	0.295
BW, g	697.3±108.7	693.6±103.9	0.717	815.5±118.2	780.6±137.0	0.005	919.4±141.9	868.5±164.2	<0.001	1032.9±163.1	983.1±199.0	<0.001	1163.6±171.0	1049.8±226.0	<0.001
Sex, male (%)	31(41)	101(54)	0.047	86(49)	170(59)	0.049	135(47)	166(52)	0.236	226(52)	171(56)	0.371	336(50)	164(56)	0.059
Surfactant use (%)	76(100)	186(100)	NA	172(99)	289(100)	0.068	284(100)	315(99)	0.371	413(95)	302(98)	0.051	642(95)	281(96)	0.305
Surfactant at DR (%)	57(75)	124(67)	0.185	121(70)	182(63)	0.123	174(62)	177(56)	0.189	206(50)	134(44)	0.145	236(67)	103(37)	0.976
Cesarean section (%)	54(71)	131(70)	0.920	127(73)	207(72)	0.752	204(72)	239(75)	0.320	321(74)	226(73)	0.817	517(76)	241(83)	0.030
Multiple births (%)	21(27)	66(36)	0.247	63(36)	85(29)	0.062	81(29)	87(27)	0.532	128(30)	92(30)	0.526	218(32)	91(31)	0.825
Antenatal Steroid (%)	67(88)	156(84)	0.376	141(81)	231(19)	0.996	245(87)	262(83)	0.239	369(86)	258(84)	0.625	574(85)	240(83)	0.405
Oligohydroamnios (%)	7(10)	26(15)	0.340	25(16)	34(14)	0.523	34(13)	52(18)	0.112	48(12)	34(12)	0.961	77(12)	43(16)	0.139

Abbreviations: NICU, neonatal intensive care unit; GA, gestational age; BW, birth weight; DR, delivery room

*A stands for infants with no or mild bronchopulmonary dysplasia

** B stands for infants with moderate or severe bronchopulmonary dysplasia

Regarding the neonatal factors, surfactant administration was performed in almost all preterm infants at less than 29 weeks GA and there was no significant difference between any of the GA groups in terms of prophylactic or therapeutic use. The frequencies of surfactant administration in the delivery room just after birth were not significantly different between the no/mild and moderate/severe BPD groups in any GA groups (Table 2).

Among infants delivered at 24 weeks GA, none of the risk factors except hypotension (36% *vs* 53%, *P* = 0.009) were significantly associated with BPD severity. Moreover, there was no significant difference in hypotension between the no/mild and the moderate/severe groups in the multivariate logistic analysis (*P* = 0.113) (Table 3). Among infants delivered at 25 weeks GA, SGA (*P* = 0.006), pulmonary hemorrhage (*P* = 0.004), tPDA (*P* = 0.002), hypotension (*P* < 0.001), IVH (*P* = 0.001), PVL (*P* = 0.001), and late-onset sepsis (*P* < 0.001) were significantly associated with severity in the univariate analysis. However, in multivariate logistic analysis, SGA (OR 2.484, 95% CI 1.080–6.052), tPDA (OR 1.873, 95% CI 1.132–3.097), hypotension (OR 2.118, 95% CI 1.263–3.553), and late-onset sepsis (OR 2.080, 95% CI 1.207–3.583) showed strong associations with an increased risk of severe BPD (Table 3). Among infants delivered at 26 weeks GA, all factors except NEC were significantly associated with severity in the univariate analysis, but after the multivariate analysis, tPDA (OR 2.078, 95% CI 1.390–3.105), hypotension (OR 1.917, 95% CI 1.280–2.872), and late-onset sepsis (OR 1.571, 95% CI 1.030–2.397) demonstrated significant differences (Table 3). For SGA, the adjusted OR was 0.897 (95% CI 0.810–0.982), which might be due to the significant difference in gestational age (*P* = 0.004) together with birth weight (*P* < 0.001) between the no/mild group (A) and the moderate/severe group (B) of infants deliver at 26 weeks GA, as shown in Table 2. Both factors seemed to have influenced each other.

**Table 3:** Comparison of neonatal factors of infants who survived during NICU stay between no/mild versus moderate/severe BPD (n = 3,039).

	24 weeks (n = 262)		25 weeks (n = 463)		26 weeks (n = 603)		27 weeks (n = 741)		28 weeks (n = 970)	
	Unadjusted	Adjusted OR*	Unadjusted	Adjusted OR* (95%CI)	Unadjusted	Adjusted OR* (95%CI)	Unadjusted	Adjusted OR* (95%CI)	Unadjusted	Adjusted OR* (95%CI)
	*P* value	(95%CI)	*P* value		*P* value		*P* value		*P* value	
SGA (<10p)	0.562	1.124 (0.307–4.124)	0.006	2.484 (1.080–6.052)	0.021	0.892 (0.810–0.982)	<0.001	3.132 (1.720–5.783)	<0.001	4.270 (2.684–7.854)
Air leak	0.295	1.423 (0.453–4.468)	0.698	1.042 (0.396–2.744)	0.037	1.617 (0.661–3.955)	<0.001	6.336 (2.247–25.319)	<0.001	2.425 (1.992–5.924)
Pulmonary hemorrhage	0.169	0.875 (0.266–2.875)	0.004	1.543 (0.518–4.592)	0.003	1.585 (0.750–3.350)	0.001	1.121 (0.442–2.846)	0.003	1.186 (0.486–2.896)
tPDA	0.519	1.584 (0.772–3.248)	0.002	1.873 (1.132–3.097)	0.004	2.078 (1.390–3.105)	<0.001	1.905 (1.289–2.704)	<0.001	1.747 (1.223–2.446)
Hypotension	0.009	1.891 (0.944–3.790)	<0.001	2.118 (1.263–3.553)	<0.001	1.917 (1.280–2.872)	<0.001	3.624 SGA, (2.322–5.826)	<0.001	2.209 (1.436–3.325)
NEC (≥ stage 2)	0.172	3.251 (0.846–12.502)	0.425	0.746 (0.310–1.797)	0.174	1.368 (0.676–2.767)	0.263	1.200 (0.569–2.534)	<0.001	1.983 (0.833–4.722)
IVH (≥ grade 3)	0.064	1.520 (0.487–4.748)	0.001	1.826 (0.835–3.991)	0.016	1.425 (0.734–2.766)	<0.001	1.645 (0.803–3.368)	0.008	1.303 (0.601–2.828)
PVL	0.558	1.520 (0.487–4.748)	0.001	1.815 (0.718–4.591)	<0.001	1.412 (0.769–2.594)	0.033	1.125 (0.595–2.130)	0.019	1.558 (0.872–2.784)
Late-onset sepsis	0.184	1.261 (0.618–2.574)	<0.001	2.080 (1.207–3.583)	0.005	1.571 (1.030–2.397)	<0.001	1.868 (1.237–2.902)	<0.001	3.099 (2.070–4.771)

Abbreviations: NICU, neonatal intensive care unit; SGA, small for gestational age; tPDA, treated patent ductus arteriosus; NEC, necrotizing enterocolitis; IVH, interventricular hemorrhage; PVL, periventricular hemorrhage

*Co-varieties included in the logistic regression included GA, multiple birth, sex, delivery, oligohydramnios, and antenatal steroids

Air leakage was a significantly strong risk factor for infants in the 27- and 28-week GA groups in both the univariate and multivariate analyses (27 weeks GA group: OR 6.336, 95% CI 2.247–25.319; 28 weeks GA group: OR 2.425, 95% CI 1.992–5.924) (Table 3). SGA, tPDA, hypotension, and late-onset sepsis were still significantly strongly associated with BPD severity even after the multivariate analysis in both the 27-week and 28-week GA groups (Table 3).

## Discussion

As mentioned earlier, data indicating the risk factors associated with the severity of BPD in infants delivered at the same GA are sparse. In this study, we demonstrated that the SGA status, hypotension, PDA, and late-onset sepsis were the most important factors for determining BPD severity. Interestingly, except for delivery type in 28 weeks GA, our study showed no statistically significant effect of major maternal factors on BPD severity in infants of the same GA.

Regarding the relationship between neonatal resuscitation and GA, infants with lower GA tended to require step-by-step resuscitation. In addition, the mortality rate was the highest among the infants delivered at 24 weeks GA (43%) and the second highest among infants delivered at 25 weeks GA (29%). This result implies the importance of appropriate, aggressive initial resuscitation for extremely premature infants to reduce the degree of severity and complications of BPD. Several large multicenter randomized trials on respiratory management after birth compared early continuous positive airway pressure (CPAP) with immediate intubation as an initial strategy [16, 17]. Each trial showed consistent BPD outcomes, demonstrating a non-significant reduction in the rate of death or BPD at 36 weeks PMA among infants treated with CPAP compared to empiric intubation and mechanical ventilation [18]. However, those trials did not specifically analyze the difference in BPD severity.

In addition to the respiratory modality, inter-center differences or different center effects could be important correlation factors for BPD development. A recent cohort study demonstrated that the rates of delivery room resuscitation between centers ranged widely from 7% to 28%; the resuscitation percentage increased with decreasing GA, which suggests specific resuscitation techniques are needed for vulnerable infants [19]. Another study mentioned the clustering effect of BPD since the incidence varied between centers even after adjustment for demographic and antenatal characteristics [20]. In this study, as a multicenter retrospective analysis of nationwide data, there should be the same kind of differences between centers. However, the KNN data do not include information about the facilities and personnel involved in resuscitation at each participating hospital; hence, we could not consider center differences.

For each GA group, infants who experienced hypotension with inotropic support less than 7 days after birth were likely to have severe BPD. It is unlikely that hypotension alone has definite causal effects on the severity of BPD because hypotension sometimes reflects multifactorial poor medical conditions. However, even apart from BPD, other studies have shown the significance of hypotension as a risk factor for mortality and adverse outcomes in VLBWIs [21]. For this reason, hypotension was not significantly different for the 24-week GA group in this study, since we excluded deaths before BPD diagnosis.

The incidence of late-onset sepsis was related to the severity of BPD in all GAs. There is much evidence that postnatal infection may contribute to the development of BPD due to the release of inflammatory mediators and to the reflux of inflammatory cells into the lung [22]. In addition to many suggested pathophysiologic links between sepsis and BPD, controlling postnatal infection seems even more important considering the epidemiological significance of late-onset sepsis. Data from the NICHD in the US reported that late-onset sepsis was diagnosed in almost 25% of VLBWIs [23]. Recently, Jung et al. reported their findings to confirm late-onset sepsis as a risk factor for BPD in ELBWIs using the KNN database [24]. Like our results, their results showed that late-onset sepsis could be a risk factor for more severe BPD. They also reported that the certain characteristics of late-onset sepsis, such as recurrent episodes before 36 weeks GA could be a risk factor for more severe BPD. However, unlike our report, they performed statistical analysis for only late-onset sepsis in the context of BPD severity [24]. Based on the previous studies above and our results, both the development and severity of BPD have a strong association with postnatal infection.

Whether PDA is a key contributor to the development of BPD is controversial among pediatricians. Despite many studies suggesting association between PDA and the development of BPD, RCTs have shown that prophylactic or early PDA treatment could not decrease the incidence of BPD or death [25]. However, recent studies have suggested that moderate-to-large PDA shunts and the duration of PDA exposure could be risk factors for the development of BPD [26, 27]. Our study reports that PDA that needs treatment could increase the risk for severe BPD. Although our research was not mainly about causation and association regarding the development of BPD, we believe our data shows that certain aspects of hemodynamically significant PDA might play causal roles in both the development and severity of BPD. Additional sophisticated studies, especially RCTs, are needed.

Our study had some unexpected results. The adjusted ORs for air leakage, pulmonary hemorrhage, and NEC for most GAs indicated that they were not risk factors for moderate/severe BPD. These results might be related to the exclusion of newborns who died before BPD diagnosis in the study protocol. Therefore, we thought that once VLBWIs were survived with aggressive resuscitation, other factors such as SGA, hypotension, and late-onset sepsis were the most important factors for worse BPD.

The current definition of BPD, including the use of supplemental O_2_ treatment at 36 weeks PMA, is a single “yes or no” definition that can be impractical for VLBWIs. Increasing evidence suggests the need for a new categorization of BPD by severity for multiple purposes, including improving the results of clinical trials and managing long-term outcomes [28]. To overcome many other limitations of the old definition, the 2016 workshop on BPD suggested refinements to definition of BPD, such as the categorization of BPD according to grade and the emphasis of severity [29]. Hence, in the present study, we tried to determine the factors in addition to low GA that might lead to more severe BPD in VLBWIs.

Even though we tried very hard to address some limitations, others were inherent to the study design and thus remained present. First, as a retrospective study, this investigation was limited by the availability of data points. We could not perform center-to-center comparisons due to the lack of limitations in the database. Second, we excluded newborns that died before BPD diagnosis from the risk factor analysis. With the inherent limitation of a retrospective multicenter study, the causes of death were not available for all of them, and the mortality rates of the 24-week group and even for 25-week GA group (29%) were high. We analyzed the initial status of all of them including the newborns who died before BPD diagnosis, to emphasize the importance of resuscitation for micro-preemies. Then, we wanted to focus on the postnatal risk factors for worse BPD. Third, the exact amount of oxygen was not calculated during the resuscitation procedures in the delivery room and the duration of oxygen use was not described as well. Our future project with KNN data might cover this aspect to obtain standardized and specific results. Fourth, clinical sepsis without culture-proven treatment can prolong the duration of ventilator care and hence partly affect the degree of BPD severity. Since few studies have attempted to identify the effect of the actual type and timing of infection on BPD, future studies can be designed to compare the relationship between BPD severity and sepsis caused by different specific types of microorganisms in infants. Fifth, variable respiratory modalities and different BPD treatment policies can affect the outcome of BPD. Hopefully, future researches with the KNN data might overcome these limitations.

In conclusion, this study revealed possible risk factors for the severity of BPD in VLBWIs by GA group. We found that extremely preterm infants delivered at less than 26 weeks GA required more aggressive resuscitation steps at the beginning to survive. Regarding all the moderate/severe BPD groups, the multivariate analysis showed that SGA, hypotension, tPDA, and late-onset sepsis were strong risk factors that could be used to predict the severity of BPD in VLBWIs. Despite the limitations of this retrospective study, we have identified possible associative factors that lead to severe BPD. Hence, these results can be helpful in managing better outcomes of BPD according to gestation in VLBWIs. Our future studies will focus on more specific resuscitation protocols for extremely premature infants, more uniform treatment guidelines for BPD and modified BPD definitions for infants who need more non-invasive ventilation.
